# Premenstrual symptoms in young adults born preterm at very low birth weight - from the Helsinki Study of Very Low Birth Weight Adults

**DOI:** 10.1186/1472-6874-11-25

**Published:** 2011-06-03

**Authors:** Sanna Mustaniemi, Marika Sipola-Leppänen, Petteri Hovi, Uriel Halbreich, Marja Vääräsmäki, Katri Räikkönen, Anu-Katriina Pesonen, Kati Heinonen, Anna-Liisa Järvenpää, Johan G Eriksson, Sture Andersson, Eero Kajantie

**Affiliations:** 1 Child and Adolescent Health and Welfare Unit, Department of Children, Young People and Families, National Institute for Health and Welfare, P.O. Box 310, Oulu 90101, Finland; 2 Department of Obstetrics and Gynaecology, Oulu University Hospital, P.O. Box 24, OYS 90024, Finland; 3 Diabetes Prevention Unit, Department of Chronic Disease Prevention, National Institute for Health and Welfare, P.O. Box 310, Oulu 90101, Finland; 4 Department of Pediatrics and Adolescence, University of Oulu, P.O. Box 5000, Oulu 90014, Finland; 5 Diabetes Prevention Unit, Department of Chronic Disease Prevention, National Institute for Health and Welfare, P.O. Box 30, Helsinki 00271, Finland; 6 Hospital for Children and Adolescents, Helsinki University Central Hospital and University of Helsinki, P.O. Box 281, HUS 00029, Finland; 7 Biobehavioral Program, School of Medicine & Biomedical Sciences, Hayes C, Suite 1, 3435 Main St., Building 5, Buffalo, NY 14214-3016, USA; 8 Department of Behavioural Sciences, University of Helsinki, P.O. Box 9, Helsinki 00014, Finland; 9 Department of General Practice and Primary Health Care, University of Helsinki, P.O Box 20, Helsinki 00014, Finland; 10 Vasa Central Hospital, Sandviksgatan 2-4, Vasa 65130, Finland; 11 Folkhälsan Research Institute, Paasikivigatan 4, Helsinki 00270, Finland; 12 Unit of General Practice, Helsinki University Central Hospital, Helsinki 00029, Finland

## Abstract

**Background:**

Clinically significant premenstrual symptoms are common among young women. Premenstrual syndrome (PMS) is characterized by emotional, behavioural and physical symptoms that consistently occur during the luteal phase of the menstrual cycle. Premenstrual dysphoric disorder (PMDD) is a severe form of PMS. Individual variation in stress responsiveness may be involved in the pathophysiology of premenstrual symptoms. Preterm birth at very low birth weight (VLBW, < 1500g) has a multitude of consequences that extend to adult life, including altered stress responsiveness which could affect the prevalence of premenstrual symptoms.

**Methods:**

In this cohort study, we compared 75 VLBW women with 95 women born at term (mean age 22.5). We used a standardized retrospective questionnaire assessing the presence and severity of a variety of symptoms before and after menses. The symptom scores were used both as continuous and as dichotomized variables, with cutoffs based on DSM-IV criteria for PMDD and ACOG criteria for PMS, except prospective daily ratings could not be used. We used multiple linear and logistic regression to adjust for confounders.

**Results:**

There was no difference in the continuous symptom score before menses (mean difference VLBW-term -18.3%, 95% confidence interval -37.9 to 7.5%) or after menses. The prevalence of premenstrual symptoms causing severe impairment to daily life was 13.3% for VLBW women and 14.7% for control women. For PMDD, it was 8.0% and 4.2%, and for PMS, 12.0% and 11.6%, respectively. These differences were not statistically significant (p > 0.1).

**Conclusion:**

Our findings suggest that the severity of premenstrual symptoms and the prevalence of PMDD and PMS among young women born preterm at VLBW is not higher than among those born at term.

## Background

It is now widely accepted that several common adult diseases have their origins during the fetal period and infancy. Although there is some heterogeneity between studies, adults born with low birth weight show increased risk of cardiovascular disease [1,2], type 2 diabetes [3] and depression [4]. Recent studies by us [5,6] and others [7-9] suggest particularly high levels of risk factors for these disorders in young adults born preterm at very low birth weight (VLBW, < 1500g).

Mechanisms that link early life events with adult disease remain poorly known. Early life programming of endocrine and other physiological responses to stress may have an important role to play [10]. There is emerging evidence that preterm birth may affect stress responsiveness in later life, in particular the function of the hypothalamic-pituitary-adrenal (HPA) axis [10-12].

Up to 30% of women of reproductive age report premenstrual symptoms severe enough to warrant diagnosis of premenstrual syndrome (PMS) [13,14]. PMS is characterized by cyclic and recurrent emotional, behavioural and / or physical symptoms which occur during the late luteal phase, remit within days of menses and are associated with suffering and impairment premestrually [14]. Premenstrual dysphoric disorder (PMDD) is a severe form of PMS characterized by mood, behavioural and cognitive complaints which seriously interfere with relationships, social activities and work. The estimated prevalence of PMDD is 3-8% [15]. The burden of PMDD during the late luteal phase may reach the same level as that of major dysphoric disorders.

Premenstrual disorders and symptoms are complex phenomena which despite decades of research remain poorly understood. Hormonal systems, in particular the HPA axis, are believed to be involved [16-18], and the prevailing view is that women with PMS are more sensitive to essentially normal hormonal swifts. Important determinants predicting how a woman will experience premenstrual symptoms are also believed to include individual differences in response to stress and adverse events earlier in life [19,20]. While lifecourse studies have thus far focused on events such as sexual abuse [21], adverse events could as well be biological and occur early in life: infants born preterm at VLBW experience after birth a period of immaturity-associated illness characterised by inadequate nutrition, impaired growth and deprivation from normal parental attachment, and the consequences of this period on hormonal function and stress responsiveness extend to adult life. With this background, we hypothesized that young adult women born at very low birth weight have a higher degree of premenstrual symptoms than their peers born at term.

## Methods

### Participants

The participants were a subgroup of the Helsinki Study of Very Low Birth Weight Adults, which has been described in detail [5,6]. Briefly, the original study cohort comprised 335 consecutive VLBW (< 1500g) infants (of whom 178 were women) who were born between January 1978 and December 1985 and who were discharged alive from the neonatal intensive care unit of Children's Hospital at the Helsinki University Central Hospital in Finland. For each VLBW survivor, the next term born (gestational age > 37 weeks) singleton infant of the same sex, and not small for gestational age (SGA, birth weight < -2SD) was selected to act as control (n = 367, of whom 201 women). We invited 255 VLBW (139 women) and 314 term (169 women) participants who lived in the greater Helsinki area to a clinical examination, and 166 (95 women, 68% of those invited) and 172 (103 women, 61%) agreed to participate.

The follow-up study was conducted between April 2004 and June 2005. In June 2004, the questionnaire for menstrually related disorders (described below) was included in the protocol of each participating woman who was not pregnant or lactating. Altogether 86 VLBW women and 101 women born at term completed the retrospective questionnaire. For the final analysis, we excluded 17 participants (11 VLBW) who reported having received treatment for one or more major mental disorder in the past three months (Table 1). The exclusion was done because comorbid mental disorders could impact the report of premenstrual symptoms [14]. The final analysis thus included 75 preterm born VLBW participants and 95 controls.

**Table 1:** Characteristics of the women

Characteristic	VLBW (N = 86) mean (SD)	term (N = 101) mean (SD)	P Value ^a^
**Birth**			
Birth weight, g	1125 (232)	3569 (474)	< 0.0001
Gestational age, wk	29.4 (2.4)	40.3 (1.1)	< 0.0001
Standard deviation score for birth weight	-1.35 (1.60)	0.08 (1.04)	< 0.0001
SGA^b^, n (%)	21 (24.4%)	0	< 0.0001
Maternal preeclampsia^c^, n (%)	19 (22.0%)	6 (5.9%)	0.001
Twin, n (%)	13 (15.1%)	0	< 0.0001
Triplet, n (%)	4 (4.7%)	0	< 0.0001
**Parental**			
Maternal smoking during pregnancy, n (%)	15 (17.4%)	13 (12.9%)	0.4
Highest education of either parent^d^, n (%)			0.2
Elementary	12 (14.0%)	9 (8.9%)	
High school	20 (23.3%)	16 (15.8%)	
Intermediate	32 (37.2%)	36 (35.6%)	
University	22 (25.6%)	39 (38.6%)	
**Current**			
Age, y	22.4 (2.1)	22.6 (2.3)	0.5
Height, cm	162.0 (7.6)	165.8 (13.5)	0.02
Weight, kg	58.3 (11.8)	63.8 (10.9)	0.001
BMI, kg/m^2^	22.1 (3.7)	22.7 (3.7)	0.2
Daily smoking, n (%)	19 (22.1%)	30 (29.7%)	0.2
Menarche, y	12.7 (1.5)	12.4 (1.4)	0.1
Regular menstrual cycle^e^, n (%)	51 (65.4%)	60 (65.9%)	0.9
Duration of menorrhea^f^, d	5.7 (2.8)	5.1 (1.8)	0.07
Hormonal contraception, n (%)	42 (48.8%)	56 (55.4%)	0.4
Contraceptive pills	37 (43.0%)	48 (47.5%)	
Hormone releasing intrauterine device	2 (2.3%)	2 (2.0%)	
Implantable contraceptive	0	1 (1.0%)	
Other or unknown type of hormonal contraception	3 (3.5%)	5 (5.0%)	
Treatment for mental disorders during previous three months^g^, n(%)	11 (12.8%)	6 (5.9%)	0.1
Depression	6 (7.0%)	5 (5.0%)	0.8
Anxiety or panic disorder	9 (10.5%)	5 (5.0%)	0.2
Eating disorder	3 (3.5%)	1 (1.0%)	0.2
Obsessive-compulsive disorder	0	1 (1.0%)	

a) The t-test for continuous and chi-square test for categorical variables.

b) Small for gestational age, birth weight < -2 SD.

c) Data missing for one term participant.

d) Data missing for one term participant.

e) Menstrual cycle is regular when its duration is 21 to 35 days [22]. Data missing for 8 VLBW and 10 term participants.

f) Duration of last bleeding. Data missing for 7 VLBW and 5 term participants.

g) These participants were excluded from analysis of premenstrual symptoms.

### Procedures

We used a validated, widely used questionnaire to assess self-reports of Menstrually-Related Disorders [22]. The assessment retrospectively enquires symptoms during the last three menstrual cycles, for each cycle during the week before menses (premenstrual period) and the week after menses. The 21 symptom items concern depressive symptoms, irritability, affective lability, anxiety, change in sleep, change in appetite (e.g. food cravings), decreased interest in activities, difficulty concentrating, feeling out of control or overwhelmed, lack of energy and physical symptoms such as breast tenderness and swelling.

The participant was asked to score the severity of each symptom from 1 to 5 (1 = minimal and 5 = extremely severe). As continuous outcome variables we used the sum of these scores before and after menses, plus the difference of these scores. In addition, the questionnaire includes three questions of the effect and burden of these symptoms to work, social relationships and daily activity, which were assessed separately.

As dichotomous outcomes, we used a) the score for each individual question dichotomized at 3 or more to indicate significant severe symptoms; b) whether the participant fulfils the DSM-IV-based criteria of PMDD [23]; c) whether she fulfils the ACOG (American College of Obstetricians and Gynecologists) [24] criteria for PMS, with the exception that we could not apply prospective daily ratings within this protocol. DSM-IV PMDD criteria presume a person to have at least five premenstrual symptoms (including at least one major dysphoric symptom: irritability, depressed mood, affective lability or anxiety) which seriously interfere with work, social activities and relationships. In addition, we assessed the proportion of women who reported at least one dysphoric symptom, which substantially impacts their life during the premenstrual period. ACOG PMS criteria require that a person has at least one affective (depression, angry outbursts, irritability, anxiety, confusion, social withdrawal) or somatic (breast tenderness, abdominal bloating, headache, swelling of extremities) symptom premenstrually during each of three prior menstrual cycles.

### Measures

Birth weight, gestational age and other perinatal and maternal data (smoking during pregnancy, preeclampsia) were collected from hospital records. The participants completed a wide range of questionnaires concerning their medical history, use of medications, current educational level, smoking and parental education (the highest level of current education achieved by either parent, categorized into four levels). Weight and height were measured and body mass index (BMI) was calculated. Questionnaires were completed in conjunction with a clinical examination. The study protocol was approved by the Ethics Committee for Children and Adolescents Diseases and Psychiatry at Helsinki and Uusimaa Hospital District. Each participant gave a written informed consent.

### Data analytic strategy

The study was powered with regard to differences in symptom score as continuous variables. With 80% power and alpha level of 0.05, 75 VLBW and 95 control participants allow us to detect or exclude a 0.44 SD difference. With 90% power and alpha 0.01, the corresponding difference is 0.60 SD. A total symptom score was calculated as the sum of the score for each specific symptom, with separate scores before and after menses. The total score after menses was subtracted from the total score before menses to evaluate the difference between the premenstrual period and time after menstruation. These scores were used as outcomes (with the total scores log-transformed to attain normality) together with the dichotomous outcome variables, calculated as described in the Procedures section. Differences between VLBW young women and term controls were examined by Student's t-test for continuous variables, Pearson's chi square test for dichotomous variables and multiple linear and logistic regressions to adjust for the following covariates: age, parental education, BMI, the current use of hormonal contraception, own current smoking and maternal smoking during pregnancy. Comparisons of participating women with nonparticipants regarding the duration of mechanical ventilation, the duration of oxygen administration and age at discharge were analyzed by means of the Mann-Whitney U test.

## Results

Gestational age of prematurely born VLBW participants ranged from 25.0 to 35.6 weeks and birth weights from 620 to 1490 g. The control participants' gestational ages ranged from 37.0 to 42.9 weeks and birth weights from 2560 to 4900 g. 24.4% of VLBW participants were born small for gestational age. The prevalence of maternal preeclampsia was 22.0% in the VLBW group and 5.9% in the control group (p = 0.001). 19.8% of VLBW participants were born from multiple pregnancies. At examination, the VLBW women were shorter (p = 0.02) and lighter (p = 0.001) than those born at term, but the groups were similar regarding the other baseline characteristics (Table 1).

Table 2 shows mean scores of premenstrual symptoms, the prevalences of premenstrual syndrome and premenstrual dysphoric disorder and each of its symptoms in study groups. In both groups the total sum scores before menses (VLBW: geometric mean 9.9; controls: 12.5) were higher than sum scores after menses (VLBW: 3.4; controls: 4.3). The difference in the sum score before and after menses was on average 7.6 for the VLBW group and 8.1 for the control group. There were no statistically significant differences between the groups.

**Table 2:** Premenstrual symptom scores among young women born at VLBW and their counterparts born at term

	VLBW N = 75	Term N = 95		
	VLBW Mean	Term Mean	Mean difference (95% CI)^b^	Mean difference (95% CI)^c^
Total score before menses	9.9^a^	12.5^a^	-19.4% (-38.2 to 5.0%)	-18.3% (-37.9 to 7.5%)
Total score after menses	3.4^a^	4.3^a^	-17.7% (-41.8 to 16.3%)	-14.7% (-40.5 to 22.5%)
Difference between total score before and after menses	7.6	8.1	-0.56 (-2.96 to 1.85)	-0.71 (-3.24 to 1.83)

	**N (%)**	**N (%)**	**Odds ratio (95% CI)^b^**	**Odds ratio (95% CI)^c^**

PMDD^d ^(DSM-IV)	6 (8.0)	4 (4.2)	1.97 (0.53 to 7.25)	1.82 (0.47 to 7.07)
PMDD symptoms^e^	7 (9.3)	9 (9.5)	0.97 (0.34 to 2.76)	0.99 (0.33 to 2.93)
PMS (ACOG)^f^	9 (12.0)	11 (11.6)	1.03 (0.40 to 2.65)	1.00 (0.37 to 2.67)
PMS symptoms seriously interfere with work, social activities, relationships	10 (13.3)	14 (14.7)	0.88 (0.37 to 2.12)	0.89 (0.35 to 2.23)
**Presence of each premenstrual symptom before menses**				
Depressed mood or hopelessness	8 (10.7)	4 (4.2)	2.71 (0.78 to 9.39)	2.24 (0.61 to 8.27)
Tension or anxiety	16 (21.3)	24 (25.3)	0.80 (0.39 to 1.64)	0.86 (0.40 to 1.87)
Affective lability	16 (21.3)	27 (28.4)	0.68 (0.33 to 1.39)	0.56 (0.26 to 1.19)
Irritability	13 (17.3)	24 (25.3)	0.62 (0.29 to 1.32)	0.61 (0.28 to 1.36)
Decrease interest in activities	6 (8.0)	5 (5.3)	1.56 (0.45 to 5.43)	1.51 (0.41 to 5.53)
Difficulty concentrating	6 (8.0)	5 (5.3)	1.57 (0.45 to 5.47)	1.59 (0.43 to 5.84)
Lack of energy	7 (9.3)	11 (11.6)	0.77 (0.28 to 2.10)	0.82 (0.29 to 2.34)
Change in appetite, e.g., food cravings	17 (22.7)	16 (16.8)	1.44 (0.67 to 3.09)	1.34 (0.60 to 2.99)
Change in sleep	8 (10.7)	9 (9.5)	1.13 (0.41 to 3.10)	1.00 (0.35 to 2.91)
Feeling out of control or overwhelmed	3 (4.0)	5 (5.3)	0.75 (0.17 to 3.24)	0.90 (0.19 to 4.23)
Other physical symptoms, e.g., breast tenderness, bloating	20 (26.7)	32 (33.7)	0.72 (0.37 to 1.41)	0.68 (0.34 to 1.38)

a) Geometric mean

b) Adjusted for age

c) Adjusted for age, parental education, body mass index, current use of hormonal contraception, own current smoking and maternal smoking during pregnancy.

d) The prevalence of PMDD (premenstrual dysphoric disorder) based on DSM-IV (Diagnostic and Statistical Manual of Mental Disorders) criteria

e) A person has premenstrually at least one of the following symptoms: depressed mood or hopelessness, tension or anxiety, affective lability or irritability, and the symptoms seriously interfere with work, social activities or relationships and work.

f) The prevalence of PMS (premenstrual syndrome) based on ACOG (American College of Obstetricians and Gynecologists) criteria

The prevalence of PMDD was 8.0% in the VLBW group and 4.2% in the controls, and the prevalence of PMS was 12.0% in the VLBW group and 11.6% in the controls. The most prevalent symptoms in both groups were affective lability, tension or anxiety, irritability and various physical symptoms. In addition, 9.3% of VLBW and 9.5% of term born participants reported at least one dysphoric symptom which substantially impacts their life during the premenstrual period. 13.3% of VLBW participants and 14.7% of controls reported that premenstrual symptoms seriously interfere with functioning at work, or with family and social relationships. As the odds ratios in Table 2 demonstrate, none of these differences was statistically significant. We also compared the prevalence of each symptom separately; again there were no statistically significant differences.

To assess possible associations with perinatal and neonatal conditions, we performed a range of analyses within the VLBW group. We compared VLBW women born SGA with VLBW women born AGA (appropriate for gestational age); those exposed to maternal preeclampsia with those not exposed; and those who developed bronchopulmonary dysplasia with those who did not. There were no differences in the total symptom scores and in the prevalence of PMDD or PMS. Neither were these outcomes associated with the duration of mechanical ventilation, the duration of oxygen administration and age at discharge.

As compared with non-participating women (= women who were invited to the clinical examination but who did not complete the premenstrual symptoms questionnaire), women who participated in the present study have similar baseline characteristics including means of birth weight (SD), gestational age, the duration of mechanical ventilation, the duration of oxygen administration and age at discharge; and prevalences of SGA, maternal preeclampsia, bronchopulmonary dysplasia and cerebral palsy diagnosed at 15 months of age (p values > 0.089).

As in our previous publications based on the same cohort [5,6], we reanalyzed the data after the exclusion of 10 participants with cerebral palsy (n = 8) or blindness (n = 2). The results were similar. An additional reanalysis was made after including the 17 participants who had originally been excluded because of a comorbid mental disorder. Again, the results were little changed.

## Discussion

We hypothesized that young adult women born at VLBW have a higher degree of premenstrual symptoms than their peers born at term. Although we used a detailed and widely used symptoms score questionnaire [22] which is intended to detect even subtle differences below the threshold of a clinically defined disorder, we could find no differences between the VLBW and control women by any applied analytical method with or without adjustment for covariates such as body size, socio-economic status, smoking and use of hormonal contraception. Although our study was not powered to detect small differences in clinical disorders, the prevalences of PMDD (8.0% in the VLBW and 4.2% in the control group) and PMS (12.0% in VLBW, 11.6% in controls) were consistent with previous reports [15,17] and any substantial excess of these disorders in adult women born at VLBW is unlikely.

Premenstrual symptoms and syndromes may be clinically relevant and cause serious impairment even when DSM-IV criteria are not met [15]. These symptoms cause severe impairment in work, social activities and relationships among a significant group of young women. In the current study, the percentages of women reporting impairment were 13.3% in the VLBW group and 14.7% in the control group. This is in accordance with estimated earlier percentages which have varied between 13-18% [15].

With the exception a few studies focusing upon HPA axis, there are little published data on the effect of preterm birth on hormonal axes including the hypothalamic-pituitary-gonadal (HPG) axis. The little data that exist suggest that both girls and boys born at VLBW have an earlier age at pubertal growth spurt than those born at term [25]. Contrary to this, large register studies suggest lower rates of reproduction [26,27] in women and men born severely preterm. This may, however, be due to personality traits leading to delays of starting romantic relationships [28] rather than differences in gonadal endocrine function which to our knowledge has not been studied.

As to the individual differences in physiological stress responsiveness, most studies have focused on the HPA axis, but published results are not consistent. In a study of 36 participants aged 8-14 years, Buske-Kirschbaum et al. (2007) reported higher morning cortisol levels in former preterm children but found no difference in HPA axis response to psychosocial stress. Preliminary findings from a subgroup of women and men (53 VLBW and 42 term) from our study cohort suggest decreased HPA axis responsiveness to stress [12]. Nevertheless, several observations may support the role of normal variation in HPA axis function in the pathophysiology of PMS. For example, cortisol levels before menstruation were lower in women who suffer from depressive symptoms premenstrually [16]. In another study women with PMS failed to show the normal increased HPA axis response to exercise during the luteal phase and showed an abnormal reaction of HPA axis to progesterone [18]. Our findings suggest that differences in hormonal functions and physiological stress responsiveness between women born at VLBW and those born at term, if they exist, are not reflected in the prevalence of reported premenstrual symptoms.

The main limitations of the study were the relatively small study population, resulting in limited power especially in sub-group analyses. However, it is of note that our sample size was larger than in most previous endocrine- and stress-related studies in people born preterm [11,12]. Moreover, as prospective daily ratings were not possible to realize, we obtained a retrospective report of the most recent 3 menstrual cycles. Retrospective self-report is not as reliable a method to reflect daily symptom experience as are prospective daily ratings, which also are the standard requirement for the accurate diagnosis of PMS or PMDD [29]. Therefore our finding of no difference in the prevalence of reported PMDD and PMS should be treated with caution.

## Conclusions

In conclusion, premenstrual symptoms are common and cause significant impairment in the daily life of many young women. However, women born at VLBW seem not to suffer from premenstrual symptoms more than their peers born at term.

## Competing interests

The authors declare that they have no competing interests.

## Authors' contributions

SM analysed data, wrote the first draft of the manuscript and coordinated other authors' contributions together with MSL and EK. MSL assisted with data analysis, contributed to manuscript redrafts and coordinated other authors' contributions together with SM and EK.

PH was a coordinator of the collection of clinical and questionnaire data, cleaned data, acquired funding and contributed to redrafts. UH contributed to conceptualization and interpretation of data as well as manuscript redrafts. MV supervised manuscript writing, acquired funding and contributed to redrafts. KR, AKP and KH collected questionnaire data and contributed to redrafts. ALJ established the study cohort, collected neonatal data and contributed to redrafts. JGE collected clinical data, acquired funding and contributed to redrafts. SA supervised clinical data collection, acquired funding and contributed to redrafts. EK supervised clinical and questionnaire data collection, data analysis and manuscript writing, acquired funding, contributed to redrafts and coordinated other authors' contributions together with SM and MSL. All authors read and approved the final manuscript.

## Pre-publication history

The pre-publication history for this paper can be accessed here:

http://www.biomedcentral.com/1472-6874/11/25/prepub
